# A snapshot of consumer engagement in clinical trials in Australia: results of a national survey of clinical trial networks and research organisations

**DOI:** 10.1186/s40900-022-00338-w

**Published:** 2022-02-05

**Authors:** Anne McKenzie, Janelle Bowden, John R. Zalcberg, Karena Conroy, Julia Fallon-Ferguson, Shilpanjali Jesudason, James Ansell, Ania Anderst, Nicola Straiton

**Affiliations:** 1 Consumer Engagement, Telethon Kids Institute, Perth Children’s Hospital, Nedlands, WA 6009 Australia; 2 AccessCR Pty Ltd, Gordon, NSW 2072 Australia; 3 School of Public Health and Preventive Medicine, Monash University, Melbourne, VIC 3004 Australia; 4 Department of Medical Oncology, Alfred Health, Melbourne, VIC 3004 Australia; 5 Consumer & Community Involvement Program, The George Institute for Global Health, Newtown, NSW 2042 Australia; 6 Sydney Research, Sydney Local Health District, Camperdown, NSW 2050 Australia; 7 School of Population Health, Curtin University, Perth, WA 6845 Australia; 8 Australian and New Zealand Alliance for Cardiovascular Trials, Perth, WA 6845 Australia; 9 Central Northern Adelaide Renal and Transplantation Service, Adelaide, SA 5000 Australia; 10 Faculty of Health and Medical Science, University of Adelaide, Adelaide, SA 5000 Australia; 11 Consumers Health Forum of Australia, Deakin, ACT 2600 Australia; 12 The George Institute for Global Health, Newton, NSW 2042 Australia; 13 Australian Clinical Trials Alliance, Melbourne, VIC 3205 Australia; 14 University of Sydney, Sydney, NSW 2006 Australia

**Keywords:** Clinical trials, Consumer, Community, Involvement, Research, Australia, Network, Patient and public involvement (PPI)

## Abstract

**Background:**

Little is known about the extent, perceptions or experiences of consumers involved in clinical trials across Australia. The purpose of this National study was to better understand the activity and perceptions of clinical trial networks (CTNs), research co-ordinating centres and their consumers, around consumer involvement in clinical trials.

**Methods:**

CTNs and research co-ordinating centres who were members of the Australian Clinical Trials Alliance (ACTA), and consumers involved in the activities of these organisations, were invited to participate in online surveys. Surveys were completed between April and September 2018.

**Results:**

80 respondents completed the surveys in full: 25 of 34 CTNs, 5 from 15 research co-ordinating centres, and included research investigators of 13 active trials, 10 completed trials. There were 27 consumer respondents. Consumers were involved in clinical trial activities across 19/25 (76%) of CTNs and 3/5 (60%) of research co-ordinating centres. Consumers were involved at all stages of the trial cycle. Despite this, only 8/30 (27%) of research organisations provided specific training to their employees or members on consumer involvement in research, and most did not have a specific policy or process relating to conducting consumer involvement in clinical trials. At the organisation level, barriers to consumer involvement in clinical trials included being unsure how to involve consumers effectively and systematically, difficulty in accessing consumers or lack of infrastructure and resources. At the consumer level, barriers included limited understanding of their roles, a lack of resources and training. Enablers included education, training and funding for both the research sector and for consumers. Almost all consumer respondents (25/27; 92%) would recommend the consumer role to other potential consumers, stating it was a valuable experience that led to knowledge exchange and learning about the research process.

**Conclusions:**

Over the last few years, consumer involvement in clinical trials has increased in Australia, but the scope of involvement varies across different research organisations, and therapeutic areas. Consumer involvement in clinical trials is valued by most that do it, however, there are opportunities to further foster and strengthen ongoing partnerships, for example by providing practical advice for researchers on how to best engage and involve consumers.

**Supplementary Information:**

The online version contains supplementary material available at 10.1186/s40900-022-00338-w.

## Plain English summary

Consumer involvement in clinical research (including clinical trials) is considered best practice in Australia, yet it is not yet standard practice. Before doing this research, we did not know the involvement, perceptions and experiences of researchers and consumers within research networks or organisations. Surveys were used to ask Australian Clinical Trials Alliance (ACTA) member organisations including their consumers, about how consumers are being involved in clinical trials. The surveys found that consumers are involved across many clinical trials conducted in Australia, and that their input is valued by researchers and research organisations. Sometimes researchers were uncertain on how to best involve consumers in the different aspects of clinical trials so their contribution was effective and added value. Consumers also expressed at times they were uncertain as to what was expected of them, and in some instances training would have been useful to help them engage more effectively. Support and training also needs to be more appropriate for people from different populations, ethnic backgrounds and cultures, including First Nations peoples. All agreed more resources, training and funding would be needed to support consumer involvement in clinical trials.

**Supplementary Information:**

The online version contains supplementary material available at 10.1186/s40900-022-00338-w.

## Background

While consumer involvement in clinical research (including trials) is considered best practice, and increasingly mandated by funders, journal and ethical approval bodies [1], it is not standard practice. Initial barriers often relate to the lack of knowledge or confidence about how to effectively involve consumers in clinical trials, and the interchangeable terminology used to describe consumer **‘**involvement**’** with passive terms like ‘participation’ were common [2]. This creates ambiguity in our understanding of what consumer involvement is, when in its truest sense, consumer involvement is decision making with or by consumers, rather than ‘to’, ‘about’ or ‘for’ them [3].

Ideally, consumers should be involved in setting research priorities, developing trial concepts, have inputting into trial design, be involved in clinical trial operational activities and contribute to the analysis, interpretation and dissemination of trial results [4, 5]. There is also a role for consumers in the governance and oversight of research at organisational and system levels, including on monitoring and steering committees [4]. Involvement should be tailored to the unique circumstances of each research project or activity.

Consumer involvement has been reported in a wide range of therapeutic areas, across a number of different jurisdictions, but despite this, researchers find navigating consumer involvement challenging [1, 2, 5–8]. Consumers may contribute in numerous ways, including direct feedback to the researchers, through meetings, or via consultation surveys [5]. Sometimes consumer involvement may occur only upon request from an external body i.e. research funders. While it is best practice to involve consumers as early as possible in the research cycle, it may occur later, once the research project is underway, or once funding is received.

Even though there is a clear ethical and moral imperative to include consumers in research, in Australia the data on consumer involvement activities and the value of involvement is not transparent (particularly in research publications), and as a result this opportunity for shared understanding and contribution is limited.

A number of barriers to consumer involvement have been identified by researchers and research organisations, including time, resources, organisational and policy barriers, finding the ‘right’ people, attitudes and balance of power, fear of ‘scope creep’ (changing requirements caused by key stakeholders), managing expectations and roles, capacity and confidentiality [9]. In addition, the language used in research may be inaccessible to consumers [9], particularly for consumers from underrepresented populations such as First Nations or those from culturally and linguistically diverse backgrounds.

While the UK, USA and Canada lead the way for consumer involvement in research [5], little is known about how consumers are involved at a national level in clinical trials across Australia—nor the extent of involvement, perceptions or experiences of researchers or consumers (although significant work has been undertaken in the mental health space [10]). The purpose of this research was to comprehend how clinical trial networks (CTNs) and research co-ordinating centres have been involving consumers in their operations and/or their clinical trial programs. While the benefits of investigator-led clinical trials conducted by CTNs have been reported previously [11], we aimed to understand the barriers and enablers to consumer involvement in clinical trials. We sought to identify opportunities to improve the capacity and capability of Australian researchers and research organisations to involve consumers more effectively and efficiently.

## Method

### Setting

This research was conducted in Australia by surveying the CTNs and research co-ordinating centres who were members of the Australian Clinical Trials Alliance (ACTA). Consumers involved in the activities of these organisations were also invited to participate. The research has been reported in accordance with the Guidance for Reporting Involvement of Patients and Public version 2 (GRIPP2) [12] (see Additional file 1). The authorship of this paper was based on a national working group formed by ACTA which was developed based on an open expression of interest. There are two consumer authors (AM, JB).

### Surveys

The Australian Clinical Trials Alliance (ACTA), on behalf of the investigator-initiated clinical trials sector, formed a steering group consisting of researchers, consumers, and policymakers amongst others to develop and co-design the surveys. Surveys were tailored and distinct for each of the three different cohorts participating; (i) respondents from CTNs and research co-ordinating centres; (ii) researchers involved in individual trials conducted by these organisations and (iii) consumers within these organisations. A pilot of the surveys was undertaken by two CTNs and two consumers, and feedback incorporated into the final surveys.

Each survey included a mixture of open and closed questions. The final *Clinical trial networks and research co-ordinating centres* and *Individual trials* surveys comprised of 18 questions each on plans for consumer involvement, how consumers had been involved, how consumers had been identified, what training or support researchers received, barriers, enablers, impact and value, and future involvement perspectives. The *Consumer* survey comprised of 29 questions in relation to consumer roles and level of involvement, training provision and effectiveness, time of involvement, impact and value, as well as barriers and enablers to being involved as consumers.

Copies of the survey instruments may be obtained from the corresponding author upon request.

### Sampling strategy

Between April and September 2018, links to the CTNs and research co-ordinating centres survey and individual trials surveys were sent directly to CTNs and research co-ordinating centres via email using contact details of ACTA members and additional organisations identified by the steering group members. CTNs and research co-ordinating centre employees were invited to complete the *Clinical trial networks and research co-ordinating centres* survey once, and the *Individual trials* survey twice, once for an active trial and once for a completed trial. CTNs and research co-ordinating centre respondents chose which trials they used to complete the *Individual Trials* survey. In total, these two surveys were sent to 49 investigator-led research organisations (34 different CTNs across a diverse range of clinical disciplines and 15 research co-ordinating centres).

The consumer survey was sent to the same mailing list (e.g., CTNs and research co-ordinating centres), for direct completion by consumers involved with those organisations. To maintain consumer privacy, the number of consumers to which the survey was sent was not collected. The steering group decided on this approach to ensure confidentiality and privacy for respondents. All surveys were implemented using the online SurveyMonkey tool (www.surveymonkey.com) and were open from April 2018 to October 2018.

Respondents were not mandated to answer all questions.

### Analysis

Data from each survey were downloaded from SurveyMonkey into Microsoft Excel format for analysis. Data analysis was performed by a contracted researcher and reviewed by one author (NS) and the steering group. Qualitative data were analysed by one author (AA) using thematic analysis whereby patterns of meaning were generated from the data [13]. The data was organised and coded using NVivo V12 (QRS International Pty Ltd). Quantitative data were analysed descriptively using Microsoft Excel (version 2017, Microsoft Corporation).

## Results

### Participants

In total, there were 80 respondents who completed the surveys in full. This included 25 respondents from 34 CTNs invited to participate (response rate 74%), five research co-ordinating centres from 15 invited to participate (response rate 33%). These respondents represented 13 active and 10 completed trials. Twenty-seven consumers involved in clinical trial activities also responded (the response rate is not calculable as the number of consumers approached was unknown to the authors). Respondents to the *Individual Trials* surveys covered a wide range of therapeutic areas including melanoma, oesophago-gastric cancer, rectal cancer, neonatal medicine, glioblastoma multiforme, paediatric food allergy, haematological malignancy and prostate cancer (Table 1).

**Table 1:** Demographics of respondents

	Clinical trial network survey (n = 25)	Trial survey (n = 13)	Consumer survey (n = 27)
Researcher role	–		–
CEO/Chair/director		4 (31%)	
Research manager/program manager/project officer		5 (38%)	
Principal investigator		1 (8%)	
Trial coordinator		2 (15%)	
Steering committee member		1 (8%)	
Has ever involved consumers in research?		–	–
Yes	19 (76%)		
No, and no plans at present	2 (8%)		
No, but plans in future	4 (16%)		
Method of invitation to participate as a consumer	–	–	
Direct invitation			14 (52%)
Expression of interest			7 (26%)
Other			6 (22%)
Consumer role	–	–	
Patient or carer			9 (60%)
Attendee of support group			1 (7%)
Clinical trial participant			1 (7%)
Other			4 (27%)
Not answered			12
Consumer type	–	–	
Patient, potential patient, carer or person using health service			7 (26%)
Consumer representative			9 (33%)
Both			11 (41%)
Have participated in a clinical trial	–	–	
Yes			5 (19%)
No			22 (81%)
Length of involvement in research, years, median (min, max)	–	–	4.75 (0.5 to 28)

Percentages are rounded to the nearest whole number. Missing data is excluded

### CTNs and research co-ordinating centres responses

Of the CTNs surveyed, 19/25 (76%) were already involving consumers, and 3/5 (60%) of research co-ordinating centres were already involving consumers. Most (4 of 6 not currently involving consumers, 67%) of the remaining CTNs were planning to involve consumers in the future.

### How were consumers involved?

Across the CTNs, research co-ordinating centres and active and completed clinical trials, there was evidence of consumer involvement in various stages of the research cycle. For 3 of 23 (13%) active or completed trials, consumer involvement was a funding requirement (Table 2). Consumers were involved to address a specific question or matter, or as a result of the value of involvement in previous studies. The most common activity was consumers reviewing new research proposals, specifically reviewing the relevance of a clinical trial against the unmet health need. One active trial had a consumer involved with implementing one arm of the study intervention by working with researchers to advise participation opportunities and educate the community on the aims of the research. Dissemination of results involved consumers using co-production methods (that is, jointly between researchers and consumers) to develop plain language summaries of study findings.

**Table 2:** Reasons for involving consumers in clinical trials, and whether the impact of consumer involvement was reported

	Clinical trial network (n = 25)	Active trial (n = 12)	Completed trial (n = 10)
Main reason for involving consumers in clinical trial*	–		
Funding requirement		4 (33%)	3 (30%)
Specific matter/issue that consumer involvement could address		7 (58%)	4 (40%)
Consumer involvement has been useful in previous studies		8 (67%)	5 (50%)
Other		3 (25%)	1 (10%)
Reports on the impact of consumer involvement			
Yes	3 (18%)		
No	14 (82%)		
Missing	8		

^*^Note reasons are not mutually exclusive so may sum to more than 100%. Percentages are rounded to the nearest whole number. Missing data is excluded

There were examples of consumers who had been involved with research teams in the development and design of clinical trials (early and late phases) across numerous disease areas, predominately oncology but also in perinatal, mental health and haematological studies. Several clinical trials respondents had listed a consumer as an Associate Investigator in their trial (n = 1), or the consumer held an advisory role throughout the trial (n = 9). This was not common, though some respondents expressed interest in involving consumers as investigators.

Consumers were also involved at an organisational level, with six organisations having an established consumer advisory panel or group. Consumers sat on scientific committees, trial management committees, trial steering committees, management advisory boards, or as member of the CTN or research co-ordinating centre board.

### How were consumers recruited?

Consumers were recruited through three major strategies: informal word of mouth, formal ‘call-outs’ via social media or email lists, or through connecting with external stakeholders, including state and national consumer organisations.

### Training

At an organisational (e.g. CTN, research co-ordinating centre) level, less than a third (n = 8/30; 27%) indicated that specific consumer involvement training was provided to their employees or research sector members. However, at an individual trial level, training about involvement was being provided to consumers in the majority (n = 8/13; 62%) of the active trials that responded and to a lesser extent in previously completed trials (n = 4/10, 40%).

Training for researchers and consumers was multifaceted and varied in format, from formal or informal, delivered face to face or web-based, internal or external. Some consumers enhanced their training by participation or attendance at organisational scientific committee meetings, and some through Consumer Advisory Panels (for example in formal courses or sessions, or via informal ‘buddy’ systems). Informal training includes mentorship or peer support whereby consumers ‘train’ one another, indirect training through consumer networks, and support through the research team.

Materials and resources consumers thought were most useful for enabling consumer involvement included formal training (web-based and in-person workshops). Resources included those that helped consumers to understand clinical trials, the structure of the research network, governance processes, ethics, data analysis and publication process, as well as description of their expectations as a consumer. Developing knowledge for consumers on how to contribute, or for researchers on how to involve and support consumers may take time: “*A lot of our [consumer] members have taken a couple of meetings to work out what they are going to contribute*”. This probably reflects inadequate systems in place to support and upskill researchers and consumers. One consumer respondent suggested “*A flyer or information pack that clearly outlines what consumer involvement means and what benefits it might have for the person would be useful*”, highlighting the need for consumers to have a clear understanding of their value and role.

### Meeting consumers’ needs

With regard to learning about research, consumers preferred face-to-face training, recognising digital literacy or access could be an issue. Consumers also wanted training that was tailored to people from culturally and linguistically diverse backgrounds. This would help to provide as wide a perspective as possible (via both research involvement and participation) and may improve the generalisability of the research findings.

CTNs noted that government and National Health and Medical Research Council (NHMRC) funding requirements for consumer involvement allowed allocation of resources “*Only recently have external drivers emerged such as government, NHMRC & other funder requirements. This provides increased motivation to direct scant resources to consumer involvement initiatives*”.

### Policies and processes

We found that many organisations participating in the surveys did not have a specific policy or processes relating to consumer involvement (n = 8/25; 32%), however if they did exist, it was most commonly in the form of a Committee Terms of Reference. One organisation, however, embedded consumer involvement in all Standard Operating Procedures, while another went even further and embedded involvement in its five-year strategic plan.

### What barriers are there to consumer involvement at an organisational or clinical trial level? Research organisation and consumer views.

Respondents reported that the main barriers to involving consumers at an organisational level were being ‘*unsure of how to undertake meaningful involvement (i.e., avoiding tokenism)*’ and the ‘*lack of funding*’. To a lesser extent, other barriers included ‘*difficulty accessing suitable consumers (e.g., lack of tools for identifying and engaging)*’, ‘*lack of time to involve consumers*’ and least of all ‘*consumer involvement deemed not necessary*’.

A number of barriers to consumer involvement were identified in our survey (Table 3).

**Table 3:** Trial and organisational barriers and enablers to consumer involvement

Barriers	Enablers
Lack of resources and training for researchers and consumers	Provision of resources and training, eg toolkits for consumer involvement
Lack of funding	Systematically addressing the shortage of resources
Lack of understanding on how to involve consumers at an organisational and operational level	Factoring consumer involvement related costs into research budgets
Perceived lack of evidence that consumer involvement adds value to clinical trials	Early involvement of consumers
	Engagement of more than one consumer representative
	Support of consumer champions at senior management level
	External drivers, e.g. funder requirement for consumer involvement

A lack of resources was one of the major barriers to engagement at an organisational level, with many in the sector expressing that they would involve consumers if funding was available: “*Shortage of resources is a barrier to effective consumer involvement and needs to be addressed systematically to improve consumer involvement in research across the board.*” Some expressed concern that, from an organisational level, consumer time was not being remunerated: “*Consumers are often asked to participate voluntarily, and this is not adequate in my mind. If we’re asking for expertise, we should be able to pay for it.*” Consumers agreed with this sentiment, noting that funding for internet and phone bills would have been helpful.

There was often a poor understanding of the role of the consumer, especially at the organisational level. CTNs felt this could be addressed via the “*availability of more information and resources including practical tools*”, support and training in the importance of consumer involvement, as well as senior management encouragement and understanding. Given the perceived lack of evidence for consumer involvement, “*building evidence for the impact and value of consumers will help to address the issue of researchers not understanding or appreciating the impact consumers can have.*” This evidence may also help shift the current work culture in some institutions that did not include consumer involvement as usual practice.

Education and training for researchers and research organisations were considered useful strategies to improve consumer involvement. Several respondents believed training to be one of the main facilitators of consumer involvement generally: “*Training for researchers. We all know we should be engaging consumers but how to do it effectively and not as tokenism*” and “*Providing training and support to consumers will improve [consumers’] ability to be involved in clinical trial activities.*”

### Consumer survey responses

The majority of consumers became involved (with research organisations or clinical trials) from a desire to help others in the future; having personal experience of the condition being studied; or by being involved in a charity or foundation concerned with the disease under study. Involvement often resulted from a direct invitation; in response to an expression of interest; or via referral from their treating clinician. Prior to the consumer involvement role, less than 20% (5/27) had participated in a clinical trial.

Consumers confirmed they were involved in clinical trials in a number of roles, with responses mirroring those of the research organisations; they are involved throughout the research cycle and in a range of operational and organisational level roles. Consumers surveyed had been involved for a median 4.75 years (range 3 months to 28 years), with most consumers having provided input for the last 3 to 6 years. Time spent on consumer activities was extremely variable (median 8 h per month, range 2 to 80 h per month).

The majority of consumers found their experience of involvement positive and expressed being adequately supported by research teams. Almost all respondents (25/27; 92%) would recommend the consumer role to other potential consumers, stating it was a valuable experience that led to knowledge exchange and learning about the research process. An enabler for consumers is clarity about their role and expectations from the outset.

Most consumer respondents stated that the research organisations they were involved with tried to include consumers from different cultural and language backgrounds, in their consumer involvement activities. Also, within their role as consumers working with research teams on a specific clinical trial, some had helped develop approaches and materials to facilitate recruitment of people from culturally and linguistically diverse populations. Materials and approaches used to support inclusion of consumers included ensuring availability of a interpreters, translating information, and producing posters and pamphlets that involved leaders in the community. However, some reported difficulties, including with translating material, or with not having sufficient time or resources to involve the broader population.

### The value of consumer involvement

Collectively, the survey responses indicated that consumer involvement was highly valued by the research organisations, researchers and consumers who responded to the surveys.

### CTN and research co-ordinating centre views

CTN and research co-ordinating centre responses focussed on the value of the consumer to the research itself e.g. by contributing to clinical trial design, ethics and oversight. CTN and research co-ordinating centres felt that consumer involvement serves to improve clinical trial design, with one respondent stating this was “*valued by the researchers at the Annual Scientific Meeting where researchers acknowledged the feedback from consumers and made appropriate changes to their study protocol. Consumers also provided their perspective on dissemination of study findings to the consumer audience using user-friendly approaches.*”

By providing valuable input used in trial participant information material and support in communicating the value of research patients, triallists reported “*They can raise issues or concerns from a different perspective than a clinician, research nurse, trial coordinator at site and co-ordinating centre staff. Explaining medical terms and procedures to patients is often difficult so it is great to get consumer engagement*”. Feedback from research organisations suggested that such involvement allowed rapid and effective engagement of the community when developing and conducting trials, potentially improving recruitment, retention and generalisability.

While the majority of CTN and research co-ordinating centre responses were positive, one respondent felt “*value was definitely limited. Consumer involvement was not a high priority*”. It is not clear whether value was limited because it was not considered a priority, or because it was not a priority because it was perceived that value was limited.

### Consumer views

Consumer respondents focussed on the value of their involvement in clinical trials to the patient community. Consumers valued their involvement because “*clinical trials exist for the benefit of patients. We [consumer representatives] represent their interests therefore our contributions are inestimable. Any investigator-led trial requires impartial feedback that it is well designed, maintains equipoise* [balance of interests]*, that public funding is being used effectively and that recruitment targets are achievable. We have a vested interest to ensure good quality trials will produce results that will ultimately improve standards of care and therefore improved patient outcomes.*” This concept of giving a rightful voice to the end-user was echoed by another consumer “*… because they [consumers] ensure that trials create value to consumers at the end of the day*”. They also felt their involvement would directly improve recruitment into studies. Consumer involvement reminds researchers of the “*people, families and carers who are impacted by their work. Not just data.*” It ensures researchers do not lose sight of the people they are working with or aiming to help through their research.

## Discussion

Consumer involvement in research, and particularly clinical trials, is rapidly growing across Australia, with noteworthy engagement both at a research organisation and individual study level. Research organisations existing in the investigator-led environment vary in the level of consumer involvement, range of activities and provision of support for both researchers and consumers. However, at all levels (organisation, trial, consumer) it is evident consumer involvement is valued by those that undertake it—particularly in the development, design and dissemination of clinical trial results to the broader community. Further, there are clear benefits for involving consumers [14–16]. Opportunities exist to continue to foster and strengthen active ongoing partnerships between organisations, researchers, policymakers and consumers. There remains a need to focus on providing practical education and training, adequate funding and increasing awareness and support for consumers to be involved in research. For example, while most consumers reported a high capacity to contribute to these activities, there remains an opportunity to improve and provide education and training to enhance consumer knowledge and skills required for such diverse roles. In the Australian context, more research is needed to understand the specific barriers and enablers for participation of people from underrepresented background such as First Nations peoples or those from culturally and linguistically diverse backgrounds [9, 17–20]. Developing accessible and culturally appropriate materials and training may help [9].

As has been reported previously, value was expressed differently, with CTN and research co-ordinating centre respondents focusing on the practical value that consumer involvement brings, while ‘Consumer’ respondents expressed wider principles of, and rationale for, consumer involvement [21]. Reiterating the significance of ‘nothing about us without us’ which highlights the importance of involving people with lived experiences in research [22–24]. Consumers intrinsically understand their experience adds value when the opportunity is presented – the problem is, of course, that the opportunity is not always presented.

Jurisdictions have implemented policies to ensure consumer involvement in the research sector [2, 5–8, 25–28]. This is particularly evident in the United Kingdom [2, 6, 8, 25–27], Europe, Canada, and the United States [29]; where consumers are included for moral, methodological and policy reasons [1]. This is less clear cut in Australia, where for example, although NHMRC research grants are partially assessed on how well research end-users (including consumers and community members) were involved in the development of the research to-date and what the plan is going forward, no guidance is provided on what the standard is, how to meet the criteria, and what weighting will be applied to the ‘end-user involvement’ components. While there was some evidence that consumer involvement may be on a ‘one off’, [1], we believe that consumers should be involved across the entire research lifecycle.

The timing of consumer involvement in research studies has been studied in a systematic review of oncology studies [5]. Often consumers were only involved at a single stage of the process, most commonly during the development of the research focus, during development of recruitment strategies, or development of methods. Consumer involvement was less likely during recruitment, analysis or dissemination of research results [5]. Our surveys identified that consumers are particularly interested in the latter stages of research and the potential improvements in health outcomes, highlighting the need to involve consumers in all aspects of the research continuum.

Barriers to consumer involvement reported in our study were broadly reflective of other Australian studies, including practical and time constraints, a lack of understanding of consumer roles and responsibilities, difficulties in accessing consumers, costs, negative or complacent attitudes of research staff, feedback requirements, and difficulty working with consumers, attitudes of balance and power, fear of scope creep, and managing expectations [9, 30, 31]. Addressing all of these barriers would likely help shift work culture, and increase the likelihood of consumer involvement in clinical research as standard practice.

Consumer involvement in clinical research design has been shown to improve enrolment and retention of clinical trial participants, especially where consumers involved have lived experience of the health condition under study; with one systematic review suggesting consumer involvement increases patient enrolment by 16% [7]; a finding that has been supported by others [28]. In a resource-constrained context, where recruitment of patients can often be difficult, this seems to be an immediate benefit of consumer involvement in study design; especially in traditionally hard to recruit areas, such as studies in mental health [16]. Apart from the many other advantages, consumer involvement also ensures that clinical trial endpoints are relevant for patients [6].

This study has some limitations. While the survey was developed through extensive consultation among the ACTA steering group (range of diverse stakeholders—including consumers), there are viewpoints that may not have been captured given the steering committee may not have been the perspective of all the intended participants. The overall sample size for CTNs and research co-ordinating centres was small, which may reflect limited consumer involvement in this space. However, the high response rate from CTNs enabled a good representation of consumer involvement activity. CTNs and research co-ordinating centres self-selected the individual trials for which they completed the survey, and as such, the representativeness of those trials to all trials in Australia is unknown. The responses from consumers provided further insight that would not have been evident from the responses provided by the wider research sector. Some respondents did not answer all survey questions resulting in partial missing data—and the reasons for missing data, and patterns of missingness were not further explored. This survey did not include clinical quality registries (organisations that monitor the quality of health care including appropriateness and effectiveness within specific clinical domains), some of which may be engaged in registry-based clinical trials with or without partnership with trials CTNs. Investigating the challenges and opportunities in relation to consumer involvement activity within national registries is an important piece of future research. Lastly, those who took part in this research were likely people who were already involving consumers and already valued this as a research activity, and thus may not be representative of all who undertake research.

Readers are invited to access ACTA’s Consumer Involvement and Engagement Toolkit via https://involvementtoolkit.clinicaltrialsalliance.org.au to access a range of practical guidance and resources.

## Conclusion

Consumer involvement in clinical research (including clinical trials) is increasing in Australia, but the scope of involvement varies across different research organisations, and therapeutic areas. Nevertheless, this study demonstrates consumer involvement is highly valued by the researchers and consumers engaged. However, there is a need for increased leadership and opportunities to further foster and strengthen ongoing partnerships between researchers and consumers, for example by providing practical advice for researchers on how to best engage consumers, develop sustainable funding streams and create accessible resources to enable inclusive involvement.

## Supplementary Information

**Additional file 1. **Guidance for Reporting Involvement of Patients and Public version 2 (GRIPP2).

## Data Availability

The data for this study is not available, but the survey tool is available on request.

## Abbreviations

ACTA: Australian clinical trials alliance
CC: Co-ordinating centre
CI: Confidence interval
CTN: Clinical trial network
NHMRC: National health and medical research council
