# Supplementary material for: A snapshot of consumer engagement in clinical trials in Australia: results of a national survey of clinical trial networks and research organisations
Source: Res Involv Engagem. 2022 Feb 5;8:3. doi: 10.1186/s40900-022-00338-w (PMC8817464; doi:10.1186/s40900-022-00338-w)
Supplement: Supplementary file 1 — Additional file 1. Guidance for Reporting Involvement of Patients and Public version 2 (GRIPP2). [file 40900_2022_338_MOESM1_ESM.docx]

GRIPP 2 Short Form

| **Section and topic** | **Item** | **Reported on page No** |
| --- | --- | --- |
| 1: Aim | Report the aim of PPI in the study | 3 and 6 |
| 2: Methods | Provide a clear description of the methods used for PPI in the study | 6-8 |
| 3: Study results | Outcomes—Report the results of PPI in the study, including both positive and negative outcomes | 12-14 |
| 4: Discussion and conclusions | Outcomes—Comment on the extent to which PPI influenced the study overall. Describe positive and negative effects | 14-17 |
| 5: Reflections/critical perspective | Comment critically on the study, reflecting on the things that went well and those that did not, so others can learn from this experience | 16 |
